# Prominin-1 and Retinal Degenerative Disorders: Expanding the Biology from Photoreceptors to the Retinal Pigment Epithelium

**DOI:** 10.3390/biom16050635

**Published:** 2026-04-24

**Authors:** Sujoy Bhattacharya, Caitlin Ang, Megan Soucy, Stephen H. Tsang, Edward Chaum

**Affiliations:** 1 Department of Ophthalmology and Visual Sciences, Vanderbilt Eye Institute, Vanderbilt University Medical Center, Nashville, TN 37232, USA; echaum@gmail.com; 2 Barnard College of Columbia University, New York, NY 10027, USA; cma2240@barnard.edu; 3 Departments of Pathology, Cell Biology, and Ophthalmology, Irving Medical Center, Columbia University, New York, NY 10032, USA; mes2350@cumc.columbia.edu (M.S.); sht2@cumc.columbia.edu (S.H.T.); 4 Department of Biomedical Engineering, Columbia University, New York, NY 10032, USA

**Keywords:** autophagy, lysosome, phagocytosis, mitochondrial function, epithelial–mesenchymal transition, partial EMT, inherited retinal dystrophy, atrophic age-related macular degeneration

## Abstract

*Prominin-1* (*Prom1*/CD133) has long been recognized as a structural determinant of photoreceptor outer segment (OS) morphogenesis, yet rapidly accumulating evidence extends its role to retinal pigment epithelium (RPE) homeostasis, encompassing autophagy–lysosomal flux, outer segment phagocytosis, mitochondrial function, and regulation of inflammatory stress. This review synthesizes mechanistic and transcriptomic insights that position PROM1 as a central regulator of photoreceptor and RPE integrity, reframing *Prom1* disease as a multi-compartment retinal disorder relevant to both inherited retinal dystrophies (IRDs) and atrophic age-related macular degeneration (aAMD). We develop a dual-axis conceptual model in which Prom1 dysfunction can initiate pathology in either the photoreceptors (OS morphogenesis failure) or the RPE, including impaired autophagic flux, lysosomal activity, defective phagocytosis, and Epithelial-Mesenchymal Transition (EMT)-like de-differentiation, with secondary cross-compartmental degeneration. Clinically, autosomal-dominant missense variants associate with macular or cone-rod dystrophy, whereas biallelic truncating/splice-site mutations drive early-onset rod–cone disease and panretinal/RPE atrophy, illustrating genotype–phenotype diversity. By integrating recent high-resolution transcriptomic data from Prom1-deficient RPE cells with long-standing insights into photoreceptor biology, we highlight converging pathways of degeneration that challenge a photoreceptor-centric view and unify disparate phenotypes within a single molecular framework. These insights broaden the therapeutic landscape, advancing gene augmentation and pathway-targeted strategies to preserve RPE integrity, sustain photoreceptor function, and modify disease course in *PROM1*-associated IRDs and atrophic AMD.

## 1. Introduction

Retinal degenerations, including inherited retinal dystrophies (IRDs) and age-related macular degeneration (AMD), are leading causes of vision loss worldwide [1,2]. The majority of IRDs are traditionally photoreceptor-centric diseases, including rod-cone dystrophies and cone-rod dystrophies, with each group characterized by a primary loss of either rods or cones and a secondary loss of other cell types, including RPE [3,4]. However, several studies have identified the retinal pigment epithelium (RPE) as the primary site of pathology in several IRDs, including Bestrophinopathies (caused by mutations in the *BEST1* gene), X-linked recessive Choroideremia (caused by mutations in the *CHM* gene), and Leber congenital amaurosis (caused by mutations in the *RPE65* gene) [5,6,7]. The RPE involvement in these IRDs shows significant phenotypic similarities to RPE degeneration in atrophic age-related macular degeneration (aAMD), which is characterized by progressive RPE dysfunction, photoreceptor cell death, and macular atrophy, suggesting that understanding the genetics and mechanisms of inherited macular degenerations may inform our understanding of aAMD [8]. A growing consensus positions the retinal pigment epithelium (RPE) as a pivotal focus of disease initiation and progression because it sustains photoreceptors through daily phagocytosis of shed photoreceptor outer segments (POSs), autophagy-lysosomal clearance of debris, mitochondrial quality control, and maintenance of epithelial polarity and barrier integrity [9]. Failures in these RPE processes lead to secondary photoreceptor death, geographic atrophy, and progressive vision loss, underscoring the centrality of RPE biology to retinal health [10]. Genetic defects affecting RPE pathways can impair photoreceptor survival well before RPE loss becomes evident, underscoring the critical need to decipher IRD pathophysiology to develop precise therapeutic interventions [11].

Since its discovery as a pentaspan transmembrane glycoprotein, enriched on plasma-membrane protrusions, *Prominin-1* (*Prom1/CD133*) has occupied a distinctive niche in retinal biology [12,13,14]. *Prom1* displays an altered splicing pattern during human retinal development, consisting of increased exon 4 inclusion and exon 25 skipping. In the mature retina, exon 4 skipping disrupts the extracellular domain of *Prom1*, producing an unstable, mislocalized protein that cannot reach the photoreceptor cilium [15]. This reduction in functional Prom1 (decreased *Prom1* expression) leads to shortened photoreceptor cone outer segments (POSs), cone cilium defects, and abnormal accumulation of cone photoreceptors, suggesting *Prom1* exon 4 inclusion is essential for normal cone maturation, structure, and function, and its mis-splicing underlies *Prom1*-associated maculopathies [15,16]. Localization studies showed Prom1’s presence at the photoreceptor outer segment base, precisely where nascent discs emerge from the connecting cilium—cementing a canonical view of Prom1 as a structural architect of disc morphogenesis [12,17,18]. Consistent with this, animal models lacking *Prom1* (global *Prom1*-KO mice) exhibit defective OS membranes, progressive photoreceptor degeneration, and visual dysfunction, establishing its structural requirement in the neural retina [18,19,20]. Although the mechanisms underlying *Prom1*-mediated maintenance or development of the OS structure are unknown, previous studies have shown that loss of Prom1’s interacting partner, Proto-CADHERIN 21, a CADHERIN homolog in the retina, is a potential mechanism for defective OS development [19,21,22]. *Prom1* is necessary for maintaining the expression levels of *ABCA4* and *RDH12* in the mouse retina, supporting the idea that *Prom1* also regulates the visual cycle, particularly at the step where all-trans-retinal is reduced to all-trans-retinol, indicating that Prom1 helps the retina maintain its cleanup and recycling step in the visual cycle [23,24].

Clinical examinations reveal a more complex and broader biology. *Prom1* variants do not yield a uniform phenotype but rather a spectrum ranging from Stargardt-like macular dystrophy, bull’s-eye maculopathy, and autosomal-dominant and recessive cone-rod dystrophies (CRD) to retinitis pigmentosa (RP)-like panretinal diseases [16,21,25,26,27,28,29]. Some young individuals exhibit early RPE abnormalities, whereas older individuals show combined RPE and photoreceptor loss, suggesting that RPE pathology precedes photoreceptor damage [30]. Others show cone-predominant disease or mixed phenotypes that cannot be explained by canonical mechanisms of disk morphogenesis [31].

As high-resolution imaging modalities, such as spectral domain Optical Coherence Tomography (SD-OCT), fundus autofluorescence (FAF), transcriptomic profiling, and cell-type-specific rodent models have matured, a conceptual shift has emerged: *Prom1* should not be viewed solely through the lens of photoreceptor architecture. Instead, *Prom1* is emerging as a multifunctional regulator of cellular homeostasis across multiple retinal cell types. In particular, its role in RPE biology—including autophagy, phagocytosis of outer segments, epithelial integrity, mitochondrial dynamics, and immune signaling—has opened a new exploratory framework for both IRDs and atrophic AMD [32,33,34,35].

This review integrates genetic, clinical, mechanistic, and transcriptomic findings to advance a new model of Prom1-related disease: one in which RPE degeneration is not a secondary response to photoreceptor pathology, but a primary driver in many forms of Prom1-associated macular degeneration. This paradigm has profound implications for understanding phenotypic heterogeneity and for designing therapeutics to preserve the health of both photoreceptors and the RPE.

## 2. Genetic and Clinical Landscape of PROM1-Associated Retinal Diseases

*PROM1* mutations span missense, nonsense, frameshift, splice-site, and intronic regulatory variants. Both autosomal dominant (AD) and autosomal recessive (AR) transmission patterns are well documented [16,25,36]. One of the earliest and best-characterized autosomal-dominant missense variants, p.R373C, is associated with bull’s-eye maculopathy, characterized by macular atrophy and cone-rod dysfunction [28]. Patients harboring this mutation exhibit progressive degeneration of the outer retinal layer, affecting both cone- and rod-mediated vision [36]. In contrast, bi-allelic loss of function alleles, including splice site-variants (c.1984-1G>T) and truncations *PROM1* c.1909C>T (p.Q637), *Prom1* c.2050C>T (p.R684), are linked to bull’s eye maculopathy and early onset rod-cone disease with panretinal/RPE atrophy [16,37,38]. At the population level, *PROM1*-associated disease appears relatively rare: variant databases catalog many distinct changes, but only a fraction are clinically validated as pathogenic, and ancestry-aware comparative prevalence estimates remain limited. Recent investigations of IRD cohorts confirm that *PROM1* mutations, although relatively rare (1–2% of IRDs), are drivers of macular dystrophy, cone-rod dystrophy, retinitis pigmentosa, Stargardt disease type 4 (STGD4), Leber congenital amaurosis (LCA), and panretinal dystrophy, reaffirming their importance despite the phenotypic variability and heterogeneity, with the route of inheritance and variant type affecting disease severity and progression [36]. Intriguingly, cohort studies confirm the prominence of p.R373C among *PROM1*-related macular phenotypes but also reveal heterogeneity in macular and peripheral degeneration, suggesting that patients with identical variants may develop distinct disease trajectories, driven by cell-type-specific vulnerabilities, modifier alleles, and potential environmental modifiers [39].

Pathogenic *PROM1* variants include loss-of-function (LOF) nonsense, frameshift, and splice-site mutations, as well as dominant-negative missense alleles, collectively leading to macular and panretinal degeneration (Table 1). LOF variants, such as p.Tyr519*, p.Ile393Argfs*21, p.Trp452Leufs*13, p.Leu8fs*, p.Gln637*, p.Arg684*, p.Ile626fs, p>his47fs, and splice defects including c.303+2T>C and c.1142-iG>A, introduce frameshifts, * premature termination codons, or aberrant splicing. These changes lead to nonsense-mediated decay or severely truncated *PROM1*, eliminating its essential role in photoreceptor morphogenesis and disc membrane architecture. These LOF alleles eliminate essential *Prom1* functions required for photoreceptor outer segment morphogenesis, disc organization, and stabilization of membrane curvature. Their loss disrupts outer-segment renewal, leading to structural disorganization and stress in photoreceptors and RPE, culminating in progressive retinal degeneration, with disease severity depending on gene dosage (dominant haploinsufficiency vs. reverse recessive null states). Beyond the clinical manifestations already described, the severity of LOF-associated disease depends heavily on allele dosage. Homozygous or compound-heterozygous LOF alleles often produce the most severe phenotypes, including LCA or panretinal dystrophy, whereas heterozygous LOF variants can lead to dominant disease through haploinsufficiency when PROM1 expression falls below a functional threshold [40]. In contrast, dominant-negative missense variants—most notably p.Arg373Cys, p.Gly240Arg, and p.Leu245Pro—primarily yield AD macular degeneration, frequently presenting as bull’s-eye maculopathy or progressive photoreceptor/RPE degeneration. Together, these data show that the pathogenic mechanism of *PROM1* variants is strongly mutation-class-dependent: LOF mutations cause disease by disrupting essential photoreceptor structural function, whereas select missense alleles exert toxic dominant effects through defective protein interactions. Recent evidence indicates that *PROM1* variants have direct consequences for autophagy regulation. Bulk RNA-sequencing analysis in *PROM1* exon-skipped human retinal organoids shows reduced expression of autophagy-associated pathways, reflecting impaired stress-response signaling primarily within cone photoreceptors, the cell population most sensitive to *PROM1* exon 4 loss [15]. In contrast, the novel *PROM1* c.232delC frameshift variant does not simply truncate the protein analogous to exon-skipping events; rather, it produces a mutated protein that is mislocalized and exhibits reduced stability, indicating a distinct structural and trafficking defect [41]. This mislocalized variant paradoxically augments autophagy, even though apoptotic pathways remain unaffected [41]. These contrasting effects of exon skipping versus c.2321delC demonstrate that *PROM1’s* role in autophagy regulation is highly domain-specific. Different structural damages—loss of a canonical domain versus creation of a truncated, mislocalized isoform—can lead to opposite functional outcomes rather than simply LOF, indicating a more complex regulatory role than previously understood (Table 1).

**Figure 1:**
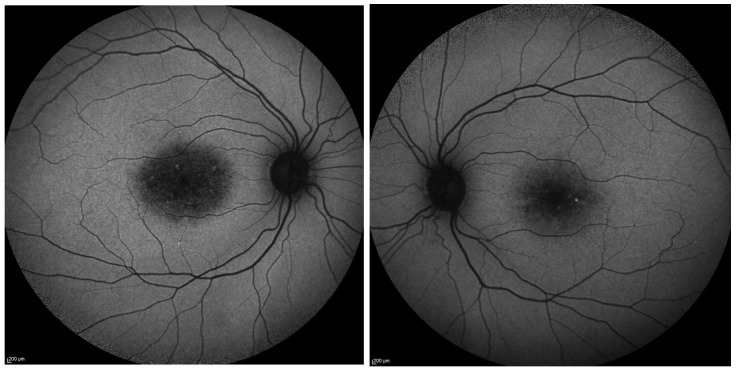
FAF images from a 40-year-old male with macular dystrophy (*PROM1* c.303+2T>C het) showing *RPE* dysfunction.

**Figure 2:**
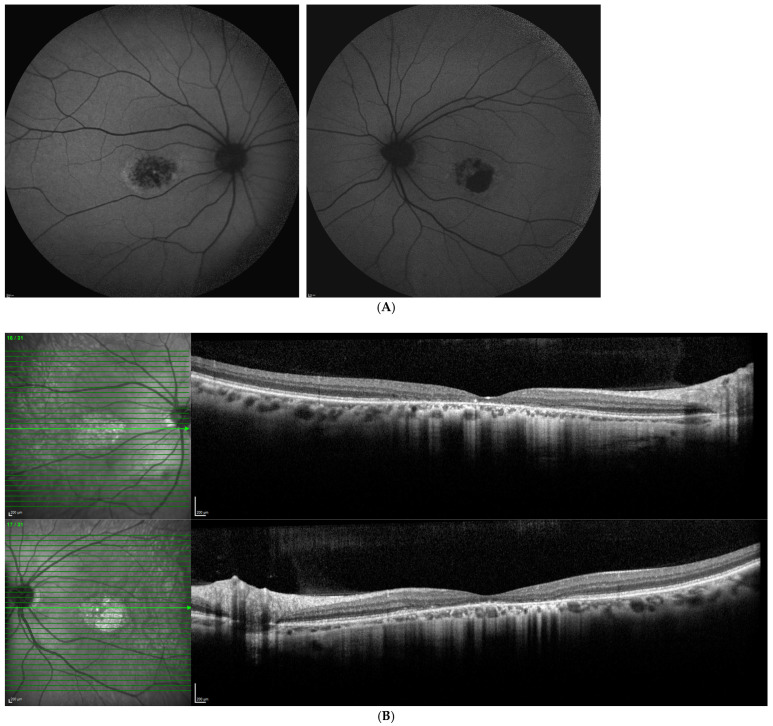
(**A**). Patient FAF images showing Stargardt disease harboring a novel *PROM1* mutation, c.276+477_304-147del (p.Lys101:Pro93del), showing Stargardt4-like disease characterized by RPE disruption. (**B**). Patient OCT images with Stargardt *PROM1* c.276+477_304-147del:p.Lys101:Pro93del mutation shows severe central macular atrophy, neuroretinal thinning, and photoreceptor layer cell loss.

FAF images from a patient with *PROM1* c.303+2T>C (heterozygous) show central macular autofluorescence abnormalities, typically presenting as a mottled hyper- and hypo-autofluorescence confined to the foveal/parafoveal region (Figure 1). This pattern reflects localized RPE dysfunction and outer photoreceptor compromise, characteristic of *PROM1*-associated macular dystrophy. The FAF paired images from a patient with *PROM1* c.276+477_304-147 del (p.Lys101:Pro93 del) display a flecked pattern of mixed hyper- and hypoautofluorescence, strongly suggestive of pattern dystrophy or Stargardt-like disease (Figure 2A). The flecks reflect lipofuscin accumulation and RPE disruption, and possibly RPE death, consistent with *PROM1*-associated STGD4-like changes. Patient OCT images with the Prom1 c.276+477_304-147del mutation showing severe central macular atrophy, neuroretinal thinning, and photoreceptor layer cell loss (Figure 2B).

Autofluorescent imaging in *PROM1* loss-of-function (p.R373C) patients reveals a characteristic macular ring of increased autofluorescence in the majority of the affected individuals [28]. The study attributes this hyper AF-ring to localized accumulation of lipofuscin within the RPE, likely reflecting impaired processing of photoreceptor OS debris or, alternatively, increased outer segment turnover—indicating that PROM1 dysfunction can produce region-specific lipofuscin buildup as a consequence of disrupted RPE homeostasis [28]. Emerging mechanistic data support this interpretation: *Prom1* loss compromises autophagy-lysosomal pathways in the RPE and diminished cathepsin-dependent degradation, thereby limiting the cell’s ability to fully digest phagocytosed OS material and predisposing to the accumulation of bisretinoid fluorophores that form lipofuscin [32,35]. In parallel, *Prom1*’s role in maintaining photoreceptor OS architecture suggests that its dysfunction may increase disc shedding or alter the composition of shed membranes, thereby further elevating the load of lipofuscin precursors reaching the RPE. Combined, these defects provide a coherent explanation for the hyper-AF rings observed clinically and link *PROM1* deficiency to the localized lipofuscin stress that precedes structural RPE compromise and the subsequent photoreceptor degeneration characteristic of Prom1-associated macular pathology.

**Figure 3:**
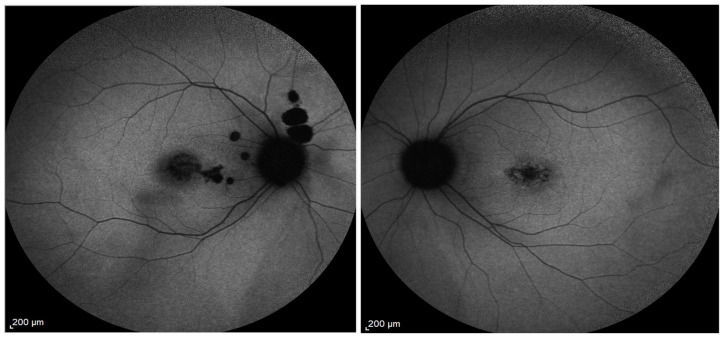
FAF images from a patient harboring *PROM1* c.1142-1G>A mutation (homozygous).

**Figure 4:**
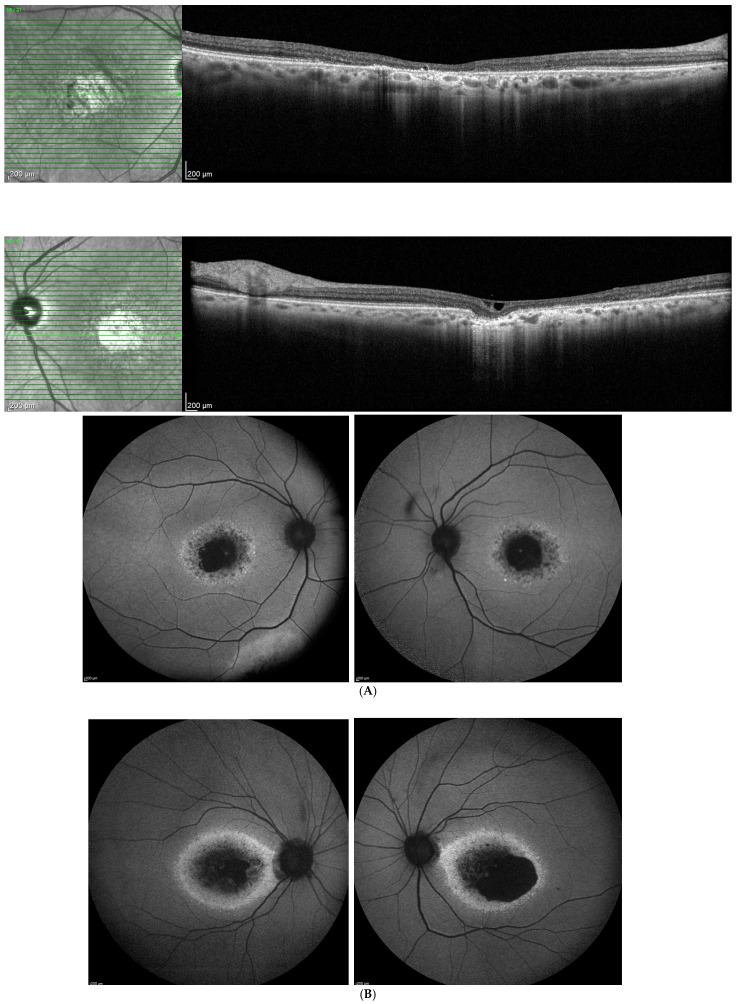
(**A**). Paired OCT and FAF images from a patient harboring a pathological PROM1 c.1117C>T p.Arg373Cys heterozygous variant showing RPE pathology. (**B**). Paired FAF images from a 67-year-old patient harboring two PROM1 variants; Prom1 c.2211+26G>A het VOUS, c.826A>G p.Thr276Ala het VOUS, phase unknown, showing RPE stress and lipofuscin accumulation.

A patient with a *PROM1* c.1142-1G>A (homozygous) shows widespread macular autofluorescence abnormalities, including patchy hypoautofluorescence and/or parafoveal hyperautofluorescence, indicating broad RPE dysfunction. This presentation aligns with severe, biallelic *PROM1*-related disease, which commonly extends beyond the central macula (Figure 3). Furthermore, the paired FAF images of a 66-year-old patient harboring the heterozygous *PROM1* c.1117C>T, p.Arg373Cys variant show a central-to-pericentral pattern of RPE dysfunction, characterized by patchy hypoautofluorescence interspersed with regions of relative hyperautofluorescence (Figure 4A). OCT imaging through the macula shows RPE structural abnormalities—the RPE layer shows irregular attenuation and focal discontinuities, accompanied by regions of ONL thinning, indicating a secondary photoreceptor loss, while inner retinal architecture remains preserved (Figure 4A). The hypoautofluorescent areas are most pronounced in the macular and perifoveal regions, consistent with focal RPE atrophy, while surrounding hyperautofluorescence zones likely reflect increased lipofuscin accumulation in stressed RPE. The transition between normal and abnormal FAF appears irregular but well demarcated, suggesting a geographic expansion of RPE pathology. A 67-year-old patient FAF images harboring two heterozygous PROM1 variants of unspecified significance (VOUS) (PROM1 c.2211+26G>A het, c.826A>G p.Thr276Ala het) early or subclinical RPE stress and lipofuscin accumulation—subtle RPE mottling characterized by variations in autofluorescence signaling without discrete geographic hypoautofluorescent lesions (Figure 4B). These cases support that pathogenic or potentially pathogenic PROM1 variants are associated with abnormal RPE lipofuscin handling, leading to pattern dystrophy and, depending on variant burden and allelic context, progression to RPE atrophy and geographic macular degeneration.

This heterogeneity suggests that Prom1 dysfunction may initiate degeneration in different cell types, depending on the mutation’s impact on protein trafficking, membrane curvature, signaling, or interactions with molecular complexes in the photoreceptor or RPE layers [31].

## 3. Prom1’s Presence and Role in the RPE

Contrary to earlier assumptions that Prom1 expression was photoreceptor-specific, our evidence from human/mouse RPE cells and in vivo studies indicates that Prom1 is expressed in the RPE and regulates homeostatic pathways central to retinal longevity [32,34,35]. Earlier studies have shown that *Prom1* is present on the apical but not the basolateral side of neuroepithelial cells in the E12 mouse embryo [51], and that Prom1 immunoreactivity was detected in the microvilli of the pigmented epithelial cells in the adult murine retina [12]. Our own studies showed that *Prom1* mRNA expression is higher in the photoreceptor-rich mouse neural retina; however, *Prom1* mRNA is detectable in situ in mouse RPE using RNAscope assays, and Prom1 protein was localized to RPE mitochondria in situ using immunogold transmission electron microscopy [33]. In addition, our studies using high-resolution immunolabeling of mouse retinal sections and transcriptomics of mouse RPE cells confirm that Prom1 plays critical roles in epithelial integrity and cellular homeostasis [35]. Consistent with these observations, we showed PROM1 expression in control and MELAS patient iPSC-derived RPE cells [52]. These observations were recently validated in a study showing *Prom1* expression and its N-glycosylation in iPSCs, iPSC-derived RPE cells, and retinal organoids [53].

Mechanistically, our studies show that in human RPE cells, Prom1 suppresses mTORC1/2 signaling, scaffolds p62-HDAC6 at nascent autophagosomes to promote autophagosome biogenesis, and traffics mature autophagosomes to fuse with lysosomes [34]. The loss of Prom1 in mouse RPE cells leads to p62 accumulation, reduction in TFEB-dependent lysosomal biogenesis, and impairment of autophagy flux [32]. In addition, Prom1 supports MerTk-dependent POS phagocytosis, linking the protein to daily clearance processes and metabolic coupling between the photoreceptors and the RPE—placing Prom1 at the intersection of proteostasis (ubiquitin-proteasome and autophagy-lysosomal pathways) and cargo handling in the RPE [35]. These abnormalities parallel mechanisms implicated in AMD pathogenesis, in which impaired autophagy contributes to drusen formation, lipofuscin accumulation, and chronic oxidative stress. Under physiological conditions, *Prom1* restrains mTORC1 signaling, thereby permitting TFEB dephosphorylation and nuclear translocation [32,34]. Active TFEB drives transcription of lysosomal hydroxylases and v-ATPase subunits, which are essential for lysosomal acidification and proteolytic capacity. In parallel, *Prom1*-dependent efficient LC3 lipidation and turnover ensure proper autophagosome maturation and timely lysosomal fusion. This coordinated autophagy-lysosome axis is critical for the daily phagocytosis and degradation of shed photoreceptor OSs—a uniquely demanding proteostatic burden borne by RPE cells. In line with an RPE-first vulnerability, mouse genetics reveal allele-dependent disease kinetics. The *Prom1*-R373C knock-in mice exhibit RPE lipofuscin-like deposits and atrophy by ~4 months, whereas the global *Prom1*-null mice preserve RPE integrity early and develop only later morphological changes [18,21]. These divergent timelines suggest that missense variants can impose a mis-trafficking burden on the RPE, whereas complete loss causes a slower, insufficiency-driven collapse of autophagy-lysosomal homeostasis, both of which converge on aberrant mTORC1-TFEB signaling and impaired degradative capacity. Hyperactive mTORC1 promotes TFEB phosphorylation and cytosolic retention, attenuating lysosomal gene expression [32]. Reduced levels of key proteases and acidification machinery compromise lysosomal competence. Simultaneously, defective LC3 processing and p62 accumulation signal impaired autophagic flux, likely exacerbated by inefficient autophagosome-lysosome fusion [32]. The resulting buildup of undigested outer segment material and lysosomal vacuoles causes chronic cellular stress, driving RPE dysfunction and, ultimately, atrophy [32,33]. This cascade provides a compelling explanation for RPE degeneration observed in *Prom1*-associated IRD. Patients with the c.1354dupT variant exhibit age-dependent phenotypes: younger patients show RPE abnormalities with preserved photoreceptors, whereas older individuals show combined RPE and photoreceptor damage, suggesting that RPE is a principal site of pathology in these patients [30]. Findings from the *Prom1*-null *Xenopus laevis* significantly reshape the longstanding view of Prom1-associated retinal pathology by demonstrating that RPE dysfunction, not photoreceptor collapse, is the earliest and primary consequence of Prom1 loss [54]. In this model, animals develop subretinal drusenoid-like deposits, progressive RPE thinning, pigment granule infiltration, and marked RPE disorganization well before photoreceptor degeneration becomes evident, indicating that *Prom1* loss primarily compromises RPE structural and metabolic integrity. These findings directly challenge the traditional paradigm that photoreceptors represent the initial targets of all pathogenic *Prom1* mutations, instead establishing a sequence in which RPE failure likely precedes and drives photoreceptor damage, at least in a subset of *Prom1*-IRDs. Consistent with these data, our own AAV2/1-mediated RPE-specific *Prom1* knockdown in mice similarly induces RPE cell death and photoreceptor degeneration, confirming that Prom1 loss acts primarily within the RPE to initiate retinal pathology [33].

### 3.1. PROM1 Regulation of Autophagosome Initiation

PROM1 is not a canonical autophagy protein, but we have evidence that it modulates autophagy at the earliest step of initiation, particularly in RPE cells. Our earlier work with human RPE showed that PROM1 regulates autophagosome initiation and maturation by acting as a cytoplasmic scaffold and directly interacting with p62 and HDAC6, both of which are crucial partners in autophagosome maturation and trafficking to lysosomes [34,55]. PROM1’s enrichment in cholesterol-rich, highly curved membrane microdomains positions it as a membrane scaffold rather than a core autophagy protein—capable of organizing signaling-permissive membranes necessary for autophagy initiation [56]. Autophagy initiation is sensitive to membrane microdomain curvature and localization of ATG14L, WIPI, and FIP200 proteins at PI3P-enriched curvature-sensing domains [57,58,59]. Therefore, loss of PROM11 is predicted to impair the spatial organization of autophagy initiation, resulting in inefficient autophagosome formation, and its localization to the RPE membrane periphery and cytosol can play essential roles in the induction of RPE autophagy.

### 3.2. Signaling Pathways Associated with PROM1-Mediated Mitophagy Regulation

While PROM1 is not a member of the core mitophagy machinery (such as PINK1, Parkin, BNIP3), it instead regulates the translation of mitochondrial stress into mitophagy. Three interlinked signaling modules exert this control: (a) AMPK-ULK1 energy-sensing axis; (b) mTORC1-TFEB lysosomal biogenesis axis; and (c) membrane microdomain organization supporting autophagosome initiation. PROM1 sets a threshold for AMPK signaling required to initiate mitophagy; without it, mitochondrial stress fails to trigger mitophagy. PROM1 suppresses aberrant mTORC1 activation in RPE cells, thereby sustaining TFEB nuclear activity, lysosomal enzyme expression, and mitophagy induction. We posit that Prom1 ensures that mitochondria delivered to lysosomes can be degraded; without it, mitophagy stalls. In RPE lacking PROM1, upstream mitophagy signaling pathways may be activated, but fail to achieve mitochondrial degradation, as PROM1-KO downregulates PINK1 gene expression, rendering cells unable to execute canonical PINK1-dependent mitochondrial ubiquitination and clearance [35].

Reframing *Prom1* as a regulator of RPE cellular homeostasis, rather than solely as a structural photoreceptor protein, opens a new therapeutic paradigm centered on restoring autophagy-lysosomal function. Instead of broad mTOR inhibition, selective attenuation of RPE mTORC1 hyperactivity may more precisely reinstate TFEB activity and lysosomal gene expression. Complementary strategies that directly enhance TFEB nuclear function, promote autophagosome-lysosome fusion, or stabilize lysosomal acidification could re-establish degradative capacity even in the setting of Prom1 deficiency. Given Prom1’s membrane-organizing properties, modulation of lipid microdomains may further normalize mTORC1 localization and signaling dynamics. Ultimately, combinatorial approaches—fine-tuning mTORC1 while augmenting TFEB and lysosomal function- may offer synergistic restoration of RPE homeostasis, shifting *Prom1* macular disease therapy from structural rescue toward targeted correction of intracellular clearance pathways.

### 3.3. Prom1 Regulation of PI3K/Akt Signaling

Cancer stem cell models provide the most detailed evidence for Prom1’s role in PI3k/Akt signaling. Prom1 directly binds to the p85 regulatory subunit of PI3K through a conserved motif in its cytoplasmic C-terminal domain. SRC-dependent phosphorylation of Prom1 at Y828 is essential for its activation, thereby promoting PI3K recruitment to the plasma membrane and subsequent Akt activation. Losing Prom1 or mutating Y828 hampers Akt phosphorylation and decreases stem cell self-renewal and tumor-forming ability, indicating that Prom1 controls PI3K activity in glioma stem cells and serves as a marker for glioma-initiating cells [60]. This Prom1-driven activation of PI3K/Akt signaling has also been shown in other tumor types, including melanoma, where Prom1/CD133-positive subpopulations display increased Akt/mTOR signaling and drug resistance [61]. Overall, these findings support a scaffold-like role for Prom1 that facilitates PI3K signaling in cancer development. In contrast to these observations, Prom1 exerts an inhibitory influence on Akt/mTOR signaling in differentiated epithelial systems. In human RPE cells, we have shown that Prom1 overexpression reduces Akt Ser473 phosphorylation (a surrogate marker of mTORC2) and S6 Ribosomal protein Ser235/236 phosphorylation (an mTORC1 downstream target), whereas Prom1 loss leads to increased Akt/mTORC1 activity, lysosomal dysfunction, and impaired autophagic flux [34]. Our recent data, using transcriptomic profiling of Prom1-KO mouse RPE cells, further show enrichment of mTORC1 signaling signatures, consistent with Akt-mTORC1 activation and metabolic stress [35]. A recent study showed enrichment of the PI3K/Akt signaling pathway in homozygous patient-specific retinal organoids expressing a pathogenic loss-of-expression Prom1 mutant, consistent with the notion that loss of Prom1 expression increases PI3K/Akt signaling [53]. These findings indicate that Prom1 does not universally activate PI3K/Akt, but rather fine-tunes pathway output in a cell-specific manner, potentially through differential binding partners or membrane microdomain organizations.

### 3.4. Prom1 Regulation of AMPK Signaling

Multiple studies show that Prom1 is a positive regulator of AMPK signaling, particularly in the context of stress-induced autophagy. We showed that STAT3-KO in glioblastoma cells increases Prom1 expression, AMPK-ULK1 signaling, and lysosomal cathepsin D processing, thereby stimulating autophagy [62]. Conversely, our data from Prom1-KO mouse RPE cells exhibited increased basal AMPK activation, evidenced by increased phosphorylation at Thr172, which was linked to inhibition of autophagy and reduced rhodopsin degradation [32]. This suggests that basal AMPK activation in Prom1-KO mouse RPE cells results from a stress response due to slowed phagocytosis and impaired lysosomal function. However, Prom1-KO reduced AMPK gene expression in mouse RPE cells (unpublished observation), suggesting that Prom1 loss induces a state of chronic stress that promotes robust upstream kinase-mediated phosphorylation of AMPK at Thr172. This post-translational activation overrides reduced AMPK abundance, resulting in enhanced AMPK signaling. Thus, decreased AMPK gene expression in Prom1-KO mouse RPE likely reflects an adaptive feedback to sustained AMPK activation rather than a reduction in pathway activity.

Our work with MELAS iPSC-derived RPE cells showed that Prom1 expression increases in RPE cells as a compensatory mechanism to maintain basal autophagic activity despite a high burden of mitochondrial damage [52]. These RPE cells with high mitochondrial heteroplasmy showed reduced basal AMPK activation, contributing to impaired autophagy, failure of mitochondrial recycling (mitophagy), and impaired lysosomal activity. These findings underscore that AMPK signaling is cell-contextually gated in RPE, with Prom1 levels dictating whether mitochondrial stress drives adaptive autophagy or lysosomal failure.

**Figure 5:**
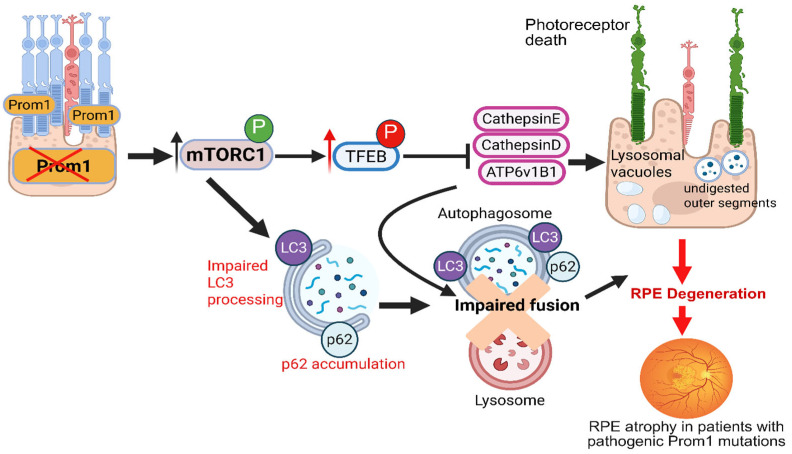
Prom1 regulates mTORC1-TFEB signaling to coordinate autophagy-lysosome function in the RPE. Loss of Prom1 function in RPE (X in red) leads to aberrant mTORC1 activation (green circle, activation), resulting in increased TFEB phosphorylation (red circle, phosphorylation of TFEB is inhibitory), thereby suppressing TFEB nuclear activity. Reduced TFEB-driven transcription diminishes expression of lysosomal genes, including Cathepsin E/D, and ATP6v1B1. Concurrently, mTORC1 hyperactivation impairs LC3 processing and promotes p62 accumulation, reflecting defective autophagic flux. Impaired autophagosome-lysosome fusion further compromises photoreceptor outer segment degradation, leading to lysosomal vacuolization and the buildup of undigested material, culminating in RPE degeneration and atrophy observed in patients with pathogenic loss-of-function Prom1 mutations. Black arrows indicate the direction of intracellular signaling between proteins, and the red thick arrows (on the right) indicate pathological processes.

Emerging evidence from our studies and those of others indicates that *Prom1* functions not only as a structural membrane organizer but also as a gatekeeper of epithelial identity in the RPE [32]. As depicted in the schematic model (Figure 5), loss of Prom1 activates mTORC1 signaling, suppresses TFEB-driven lysosomal programs, and dampens autophagic flux, collectively imposing metabolic and lysosomal stress on the RPE. In addition, transcriptome profiling of Prom1-deficient RPE cells ties Prom1 to mitochondrial homeostasis via PINK1-mediated mitophagy, oxidative phosphorylation, stress/inflammatory signaling, and junctional programs, reinforcing Prom1 as a systems-level regulator of RPE identity and metabolism [35]. Our transcriptomics studies showed significant upregulation of hallmark signatures for cell cycle transcription factors (*E2F* and *MYC* targets, *G2M* checkpoint), *mTORC1* signaling, unfolded protein response, reactive oxygen species pathway, *TNFA* signaling via *NF-kappaB*, DNA repair, and oxidative phosphorylation pathway in WT vs. *Prom1*-KO mRPE cells. Downregulated pathways included the apical junction, bile acid metabolism, and EMT pathways. Together, these observations suggest that Prom1 loss in mouse RPE drives a transcriptional reprogramming toward RPE innate immune and stress response remodeling, dampening of mitochondrial energy metabolism, and cell cycle progression, promoting a maladaptive state that compromises RPE homeostasis and resilience [35].

## 4. Role of Prom1 in RPE EMT

**Figure 6:**
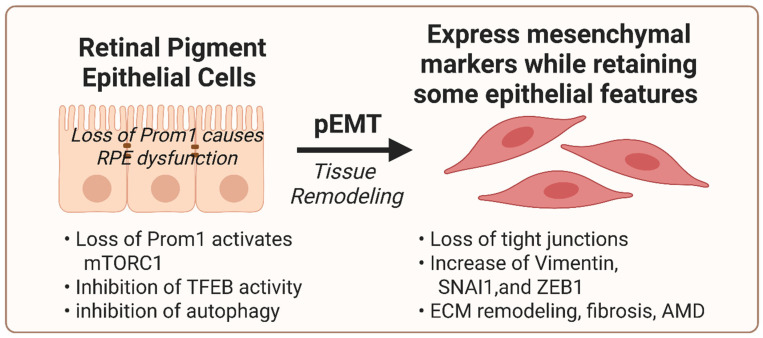
Loss of PROM1 promotes mTORC1 activation and induces partial EMT (pEMT) in the RPE. Under homeostatic conditions, Prom1 supports epithelial integrity in RPE cells. Loss of Prom1 activates mTORC1 signaling, suppresses TFEB activity, and inhibits autophagy, collectively contributing to cellular stress and phenotypic programming. RPE cells undergoing pEMT exhibit loss of tight junction integrity while retaining select epithelial characteristics. This transitional state is characterized by increased expression of mesenchymal markers, including Vimentin, SNAI1, and ZEB1, as well as ECM remodeling. The resulting tissue remodeling environment promotes fibrosis and may contribute to macular pathology, including features observed in advanced degenerative disease.

Rather than undergoing complete epithelial-mesenchymal transition (EMT), *Prom1*-deficient RPE cells adopt a partial EMT (pEMT) state characterized by loss of junctional and polarity genes, disruption of tight junctions, cytoskeletal reorganization, alongside induction of mesenchymal and stress-associated pathways, while retaining aspects of epithelial features, though clarifying spatial-temporal relationships and defining EMT endpoints remain necessary to sharpen mechanistic inference (Figure 6) [35]. Our previous data show that *Prom1* loss in mouse RPE cells correlates with decreased ZO-1 and E-CADHERIN levels and a concomitant increase in VIMENTIN, SNAI1, and ZEB-1 protein levels [32]. In addition, the nuclear localization of both SNAI1 and ZEB1 proteins increased in Prom1-KO cells, suggesting that Prom1 plays a role in the transdifferentiation of mouse RPE cells to a mesenchymal phenotype [32]. In recent transcriptomic studies, we found that *Prom1*-KO upregulated EMT-associated genes, including *Grem1*, *Pcolce2*, *Cxcl2*, *Cd44*, and *Serpine2*, whereas *Igfbp2 and Postn* were notably downregulated [35]. Although gene set enrichment analysis did not reveal broad enrichment of canonical EMT pathways, targeted analysis of individual transcripts, including upregulation of the EMT-promoting gene *Grem1* and downregulation of *Postn*, a gene critical for RPE structural integrity, supports the induction of a partial, context-specific EMT-like transcriptional program in *Prom1*-deficient RPE cells [35]. This hybrid phenotype aligns with the RPE’s response to chronic mitochondrial dysfunction and inflammatory cues, providing a mechanistic bridge between *Prom1* loss and degenerative phenotypes, including maladaptive tissue remodeling, extracellular matrix deposition, and fibrotic progression—implicated in IRDs and aAMD [63,64]. Thus, *Prom1*-regulated mitochondrial and lysosomal homeostasis, together with junctional stability, is essential to prevent pEMT-driven RPE dysfunction in retinal degenerative disease.

Conceptually, *Prom1*-associated macular disease may therefore reflect a chronic, mTORC1-driven epithelial destabilization program rather than purely degenerative cell loss. This reframing opens therapeutic opportunities to stabilize epithelial identity. Targeted suppression of mTORC1 hyperactivity, activation of autophagy, restoration of TFEB-mediated lysosomal function, or reinforcement of junctional complexes could prevent or reverse the pEMT trajectory [65,66,67]. More innovatively, therapies designed to modulate EMT plasticity, such as selective inhibition of SNAI1/ZEB1 transcriptional networks or epigenetic reprogramming strategies that reinforce epithelial chromatin states, may recalibrate RPE cells away from a fibrogenic phenotype. Combining metabolic rebalancing (mTORC1 modulation), as shown earlier to play an important role in RPE EMT in aAMD pathogenesis, with anti-fibrotic or ECM-targeted approaches could interrupt the feed-forward loop between cellular stress and tissue remodeling [66]. The mesenchymal transition of RPE cells and ECM dysfunction are two main aspects of fibrotic scar formation and are associated with impaired autophagy [68]. Mechanistically, Prom1 loss perturbs autophagy-lysosomal crosstalk and apical membrane organization, sustaining mTORC1-dependent TFEB phosphorylation, blunting lysosomal biogenesis and autophagic flux; the resulting lysosomal alkalinization and reduced cathepsin activity stabilize EMT drivers (SNAI1/ZEB1) and TGF-beta receptor signaling, while disrupted junctional maintenance (ZO-1) and RhoA-ROCK-driven cytoskeletal tension engage YAP/TAZ and integrin-FAK signaling to amplify ECM synthesis (collagen I, fibronectin, MMP/TIMP imbalance) and matrix stiffening- cardinal features of pEMT and pro-fibrotic remodeling [67,69,70,71,72,73]. Prom1’s role in photoreceptor architecture suggests that its dysfunction can lead to structural instability and may, secondarily, influence the composition and turnover dynamics of shed OS membranes, thereby altering the quality of lipid and bisretinoid precursors reaching the RPE. Accumulation of A2E-rich lipofuscin compromises lysosomal stability and activates ROS/NLRP3-linked stress responses, reinforcing mTORC1 activity and amplifying the pEMT-ECM feed-forward loop [74,75]. In this context, *Prom1* emerges not merely as a photoreceptor-associated gene but as a central regulator of epithelial stability in the macula, positing pEMT modulation as a rational and potentially disease-modifying strategy for *PROM1*-associated macular disease.

Together, genetic, clinical, mechanistic, and transcriptomic findings support a new model of *Prom1*-related disease: RPE degeneration can be a primary driver, rather than merely a secondary consequence of *Prom1*-associated retinal degeneration in a substantial subset of genotypes. This paradigm helps explain phenotypic heterogeneity across dominant missense and recessive null-like alleles and supports therapeutic strategies that preserve both photoreceptor and RPE compartments. Longitudinal imaging (SD-OCT, FAF) of patients, cell-type-specific *Prom1*-KO mouse models, identification of biomarkers, and ancestry-aware genetics will be essential to disentangle cell-of-origin trajectories across disease stages and to promote interventions that restore autophagy-lysosomal function, enhance phagocytic capacity, and stabilize mitochondrial bioenergetics in the RPE.

## 5. Prom1 IRDs and aAMD: A Shared Pathway?

Although the connection between Prom1 dysfunction and aAMD pathogenesis is presently weak, the mechanistic overlap between Prom1-IRDs and aAMD is increasingly evident. Multiple convergent pathways—impaired autophagy and lysosomal dysfunction, oxidative stress and mitochondrial dysfunction, epithelial dedifferentiation and junctional instability, and defective outer segment metabolism—are shared features of both disease contexts. Emerging in vivo studies further reinforce this connection: Prom1-null *Xenopus laevis* exhibit early RPE dysfunction, RPE thinning, and drusenoid-like deposits, demonstrating that Prom1 loss primarily destabilizes the RPE rather than initiating photoreceptor pathology. These findings collectively point to an aAMD-like endophenotype arising from Prom1 deficiency, in which early RPE destabilization preceded downstream degenerative events. Our own studies show that RPE-specific Prom1 knockdown in a mouse model causes RPE degeneration, confirming that Prom1 operates cell-autonomously within the RPE and that RPE failure is a primary driver of subsequent retinal degeneration. These insights suggest that Prom1 biology highlights aspects of aAMD pathogenesis not fully explained by aging or genetic risk alone. Loss of Prom1 function generates a molecular and metabolic environment remarkably similar to that of early aAMD—even in models lacking classical AMD susceptibility alleles—thereby strengthening the idea that Prom1-associated disease constitutes a mechanistic bridge between IRD and aAMD. An important next step is to evaluate whether this mechanistic overlap extends to human genetics. Specifically, the hypothesis that Prom1 variants contribute to aAMD susceptibility could be directly tested by screening patients with aAMD for rare or pathogenic Prom1 variants, thereby determining whether subtle reductions in Prom1 function occur more broadly in the aging retina. Additional studies are required using conditional and tissue-specific Prom1 knockouts in RPE and photoreceptors to dissect Prom1’s contribution across retinal cell types.

## 6. Future Directions and Therapeutic Implications

**Figure 7:**
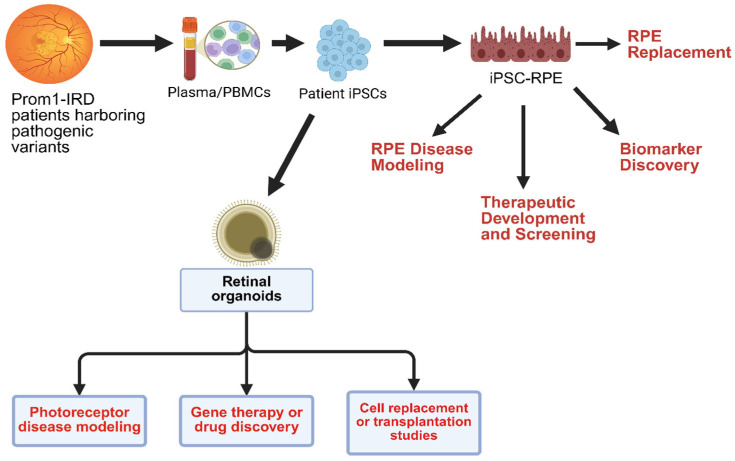
Pipeline for patient-specific translational research in Prom1 IRD: PBMC-derived patient iPSCs yield iPSC-RPE and retinal organoids that enable RPE and photoreceptor disease modeling, therapeutic screening, biomarker discovery, and cell-replacement studies, supporting single and combined-compartment interventions. Plasma (red) denotes the cell-free circulating compartment, whereas the color in PBMCs represents the mixed mononuclear immune cell population. The arrows denote the direction of biological communication and/or experimental workflow from PROM1 patients to iPSC-RPE or retinal organoids.

Prom1’s dual role in photoreceptor structure and RPE homeostasis expands therapeutic opportunities across multiple fronts, and the patient-to-pipeline depicted in Figure 7 operationalizes these opportunities in human-relevant models. PBMC-derived patient iPSCs can be used to generate iPSC-RPE and retinal organoids, enabling modeling of RPE and photoreceptor diseases, biomarker discovery, therapeutic screening, and potential RPE/photoreceptor replacement strategies (Figure 7). Together, these complementary systems provide Prom1 variant-specific readouts of efficacy and safety, allowing prioritization of single- or combined-compartment interventions.

### 6.1. Gene Therapy

AAV-based supplementation remains a compelling strategy for recessive loss-of-function variants of Prom1. In contrast, dominant-negative variants may require a combined approach—allele-specific suppression paired with gene replacement to restore functional protein expression. The patient iPSC platform can enable testing of Prom1 function rescue across iPSC-RPE (RPE-directed) and retinal organoids (photoreceptor-directed) contexts, while concurrently identifying biomarkers for translation. A photoreceptor-targeted AAV-hProm1 vector was recently used to restore Prom1 expression and OS-like structures in patient-derived retinal organoids [53]. Another study used CRISPR/Cas9-mediated correction of patient iPSCs with Prom1-IRD to genetically restore Prom1 function, showing that the gene-editing strategy could inform the design of gene-therapy studies targeting the early stages of Prom1-related IRDs [30].

### 6.2. RPE-Targeted Therapies

Given that Prom1 plays a central role in maintaining RPE metabolism, polarity, and autophagy, interventions to enhance autophagy, stabilize mitochondria, or strengthen epithelial junctions—offering mutation-agnostic strategies that may mitigate degeneration even without a direct genetic connection. Small molecules that promote mitophagy and restore lysosomal acidification may represent promising avenues for treating the downstream effects of Prom1 dysfunction.

### 6.3. Precision Modeling

Patient iPSC-derived RPE and retinal organoids enable Prom1 variant-level, human-relevant disease modeling for mechanistic dissection and high-throughput drug- and gene-therapy screening to identify mutation-specific therapeutic vulnerabilities and responses. Co-culture or interface models (organoids + RPE) further capture bidirectional pathology and help stratify therapies by allele class and initiating compartment.

### 6.4. Combined-Compartment Therapies

Because degeneration can originate in either photoreceptors or RPE, depending on variant and context, multi-compartment strategies, including concurrent photoreceptor gene rescue with RPE autophagy restoration, or staged cell-replacement (RPE monolayer and/or photoreceptor transplantation), may be essential to prevent long-term retinal architecture and visual function.

In sum, we provide a translational workflow, from patient blood draw to compartment-specific human models, that supports gene therapy/small-molecule rescue, biomarker development, and cell-replacement strategies, with the flexibility to deploy single- or dual-compartment interventions matched to Prom1-variant biology.

## 7. Conclusions

Prom1 is no longer viewed as solely a membrane-shaping protein for photoreceptor discs. It is a multi-functional regulator of cellular integrity in both photoreceptors and RPE. Its dysfunction triggers a web of pathogenic processes—structural collapse, metabolic compromise, loss of epithelial identity, mitochondrial dysfunction, and impairment of lysosomal activity—that explains the spectrum of Prom1-associated IRDs and their striking similarity to aAMD. By reframing Prom1 through a broader biological lens, we not only unify the degenerative pathways of IRDs and AMD but also identify new therapeutic opportunities. Prom1 thus stands at a critical intersection of retinal cell biology, degenerative disease mechanisms, and translational innovation.

## Figures and Tables

**Table 1:** Comprehensive Listing of Pathogenic *PROM1* Variants: Molecular Classification and Clinical Correlates.

Prom1 Variant (s)	Predicted Amino Acid Change	Genotype	Variant Effect(s)	Phenotype	Reference
c.1557C>A	p.Tyr519*	Compound Heterozygous	Nonsense	Retinal dystrophy	ClinVar RCV000504778.6 [42]
c.1177_1178delAT	p.Ile393Argfs*21	Compound Heterozygous	Frameshift	Retinal dystrophy	ClinVar RCV000504956 [31]
c.1354_1355insT (c.1354dupT) (LOF)	p.Tr452Leufs*13	Heterozygous and Homozygous	Frameshift	AR, CORD, RP, and STGD4	PMID: 38956727 [30]
c.22del	p.Leu8fs*	Heterozygous	Frameshift (LOF)	Severe retinal dystrophy	PMID: 31199449 [31]
c.436C>T	p.Arg146Ter	Heterozygous (dominant) or Homozygous (recessive)	Nonsense	AR, Retinitis Pigmentosa	ClinVar: RCV000987427.1 [29,42]
c.199C>T	p.Gln67*	Homozygous	Nonsense	Retinal dystrophy	PMID: 31199449 [31]
c.1142-1G>A	Splice acceptor site	Homozygous	Aberrant splicing	STGD4, Retinitis pigmentosa. CORD, macular dystrophy	ClinVar: RCV002497313.2 PMID: 31199449 [31]
c.1117C>T	p.Arg373Cys [AD]	Heterozygous	Missense-dominant negative	Macular and peripheral RPE degeneration	PMID: 38072963 PMID: 28840994 [26,43]
c.1901C>T c.2020C>T	p>Gln637* [AD/AR] p.Arg684* [AD/AR]	Heterozygous Homozygous	Nonsense- Truncating-LOF Nonsense- Truncating-LOF	AD- Bull’s eye maculopathy AR-panretinal dystrophy	PMID: 35951719 [37]
c.642T>A	p.Tyr214* [AD]	Heterozygous	Nonsense- Premature stop-LOF	Retinal dystrophy Best retinal disease	PMID: 26702251 [44]
c.2110C>T	p.Arg704Cys [AR]	Heterozygous	Missense	Retinal dystrophy	ClinVar: RCV001058099.8
c.303+2T>C	Splice donor	Heterozygous	Splicing abnormality, exon 4 skip- null function	Macular dystrophy; early-onset rod-cone dystrophy	PMID: 40724865 [25]
c.718G>A	p.Gly240Arg	Heterozygous	Missense	AD Bull’s eye macular dystrophy	ClinVar: RCV00036867.2, RCV000262406.5
c.400C>T	p.Arg134Cys	Heterozygous	Missense	AD Stargardt-like macular dystrophy	PMID: 31576780 [45]
c.1877_1878del	p.Ile626fs	Heterozygous	Frameshift deletion- loss of protein	Leber’s congenital amaurosis/ macular atrophy	PMID: 31836589 [46]
c.139del	p.His47fs	Heterozygous	Frameshift deletion- loss of protein	Leber’s Congenital Amaurosis/ Macular Atrophy	PMID: 31836589 [46]
c.734T>C	p.L245P	Heterozygous	Missense	Stargardt4-like macular Dystrophy	PMID: 29416601 [27]
c.1726C>T	p.Q576X	Homozygous	Missense	AR RP with macular degeneration RPE atrophy	PMID: 17605048 [29,44]
c.1841delG	p.G614Efs12X	Homozygous	Frameshift- truncated non-functional protein	AR RP with macular degeneration	PMID: 10587575 [12,44]
c.869delG	p.S290IfsX	Homozygous	Frameshift—truncated protein-LOF	AR RP with macular degeneration	PMID: 20042663 [47]
c.2321delC	p.A774Vfs*2	Heterozygous	Frameshift	AR cone-rod dystrophy	PMID: 40414337 [41]
c.2485G>A	p.D829N	Heterozygous	Missense	AD Macular Dystrophy	PMID: 28095140 [48]
c.334T>C	p.C112R	Heterozygous	Missense	AD Macular Dystrophy	PMID: 32820593 [49]
c.2327A>T	p.D776V	Homozygous	Missense	AR Macular Dystrophy	PMID: 28095140 [48]
c.7dup	p.L3Pfs28*	Compound heterozygous	Frameshift	AD Stargardt-like macular dystrophy	PMID: 26103963 [50]

* indicates a premature stop codon, causing the protein to be truncated (shortened) and often results in a non-functional protein.

## Data Availability

No new data were created or analyzed in this study.

## Abbreviations

The following abbreviations are used in this manuscript:

IRDs	Inherited Retinal Dystrophies
aAMD	Atrophic Age-related Macular Degeneration
RPE	Retinal Pigment Epithelium
mTORC1	Mammalian Target of Rapamycin Complex 1
TFEB	Transcription Factor EB
Prom1	Prominin-1 (CD133)
AD	Autosomal Dominant
AR	Autosomal Recessive
STGD4	Stargardt disease 4
EMT	Epithelial-Mesenchymal Transition
pEMT	partial EMT
ECM	Extracellular matrix
AAV	Adeno-associated virus
POS	Photoreceptor Outer Segment
LCA	Leber congenital amaurosis
CRD	Cone-rod dystrophies
RP	Retinitis Pigmentosa
BEST1	Bestrophin-1
CHM	Choroideremia
LOF	Loss-of-function
FAF	Fundus autofluorescence
SD-OCT	Spectral Domain Optical Coherence Tomography
iPSC	induced pluripotent stem cell
PBMC	Peripheral Blood Mononuclear Cell
MMP	Matrix Metalloproteinases
TIMP	Tissue Inhibitors of Metalloproteinases
MerTK	Mer proto-oncogene Tyrosine-Kinase
FAK	Focal Adhesion Kinase
ROS	Reactive Oxygen Species
NLRP3	NOD-like Receptor Protein 3
TGF-beta	Transforming Growth Factor-beta
VUS	Variant of un-specified significance
